# The Effect of Surface Acting on Job Stress and Cognitive Weariness Among Healthcare Workers During the COVID-19 Pandemic: Exploring the Role of Sense of Community

**DOI:** 10.3389/fpsyg.2022.826156

**Published:** 2022-03-10

**Authors:** Arman Sousan, Panteha Farmanesh, Pouya Zargar

**Affiliations:** Business Faculty, Girne American University, Karmi, Cyprus

**Keywords:** surface acting, job stress, cognitive weariness, spirituality, nurses, sense of community, COVID-19, Iran

## Abstract

Surface acting (SA) is a heavy emotional and cognitive task practiced by nurses, which has negative consequences on their wellbeing. The shortage of nurses along with the occurrence of the COVID-19 pandemic has worsened the situation. Based on job demands-resources (JD-R) and conservation of resources theories, this study aims to investigate the adverse impact of practicing SA and buffering effect of a sense of community (SOC) on job stress (JS) and cognitive weariness (CW) among Iranian nurses confronting COVID-19. As this study is written within the scope of Frontiers’ call for research on “Spirituality in the workplace,” the findings suggest that the aforementioned factors are in significant positive relationships. Furthermore, the empirical evidence indicates that there is a significant indirect effect of SA on CW through JS. In addition, results reveal that there is a buffering effect of SOC on the SA and JS relationship, while there is no significant moderation effect regarding the JS and cognitive weariness association. The findings of this study provide theoretical and practical implications within the scope of spirituality in the workplace.

## Introduction

Nurses experience heavy job demands due to the nature of their occupation, and during the COVID-19 pandemic, the situation has become increasingly extreme. This has become a topic of interest for scholars in the adverse consequences of demanding jobs during the COVID-19 pandemic (e.g., Hoseinabadi et al., 2020). There are numerous studies reporting disconcerting findings on negative wellbeing outcomes among nurses worldwide (e.g., Catania et al., 2021; Kackin et al., 2021), and particularly, Iran (e.g., Karimi et al., 2020; Nemati et al., 2021) confronting COVID-19. Studies present empirical evidence suggesting a global increase in the level of insomnia, suicidal behavior, post-traumatic stress disorder, and burnout among healthcare workers (HCWs) (Chirico et al., 2021b). Volunteers in the healthcare system who are in continuous and close contact with suffering patients, similar to HCWs, are at risk for burnout (Chirico et al., 2021a). Furthermore, nurses practice heavy tasks regarding emotional regulation to fulfill their job requirements due to high market competition and customer-centered services in the medical business environment (Grandey and Melloy, 2017). Similar to the current global nursing shortage (Spurlock, 2020), Iran is experiencing a serious shortage of nurses (Shamsi and Peyravi, 2020). Thus, we proposed that the findings of this study may have international implications for researchers and practitioners interested in nurses’ wellbeing.

According to *job demands-resources (JD-R)* and *conservation of resources (COR)* theories, emotional and cognitive efforts increase the perceived job demands and communal resources that contribute to higher levels of perceived job resources by individuals (Demerouti et al., 2001; Hobfoll et al., 2018). When job demand exceeds job resources, it will drive individuals to experience negative states of wellbeing through job stress (JS) (Schaufeli and Taris, 2014). *Surface acting (hereafter SA)* is the practice of emotional regulation to align felt emotions with required emotional display for conducting an occupation, which requires significant emotional and cognitive endeavors (Grandey and Melloy, 2017). *Sense of community (SOC)* is the dimension of workplace spirituality, which occurs at the group level concerning interactions among employees to evaluate the deepness of their interconnections (Milliman et al., 2003). It is a valuable communal resource for individuals to cope with stressors such as SA through perceiving spirituality (Zou and Dahling, 2017). There are studies suggesting workplace interventions as highly effective actions for improving the mental wellbeing of high-risk groups (e.g., Pieper et al., 2019; Chirico and Ferrari, 2021; Fox et al., 2021).

Surface acting is found as a destructive emotional labor strategy regarding the generation of JS (Kim, 2020) and the consequent cognitive weariness (CW) (Bakker and Heuven, 2006). While CW is one of the dimensions of burnout, reflecting the lack of focus and mental capacity due to overexposure to stressors (Melamed et al., 1999), it may lead to fatal errors among nurses, which is a critical problem and underlying concern of this study. Thus, this study aims to investigate the adverse effects of SA on nurses confronting COVID-19 regarding CW. In addition, focusing on SA and emitting deep acting will reduce the social desirability bias in nurses’ responses to this study survey, which is thoroughly elaborated in the section “Data Collection and Sampling Design.”

There are numerous studies examining the effect of individual differences in experiencing emotional labor (Kammeyer-Mueller et al., 2013). However, there are personal differences in different contexts, which remain unexplored (Zou and Dahling, 2017). This study focuses on specific aspects of workplace spirituality, namely, SOC as differences among nurses confronting COVID-19. This study aims to fill a gap in the literature regarding the moderation effect of SOC as the group-level dimension of workplace spirituality on the relationship between SA and CW through perceived JS. The study thus contributes to the extant literature regarding the negative consequences of SA and the possible buffering effect of group-level workplace spirituality. According to the aforementioned theories, evidence suggesting the shielding effect of SOC as a job resource against the adverse effect of SA as a job demand on JS and the resulting CW is expected.

## Theoretical Overview and Development of Hypotheses

### Job Demands-Resources and Conservation of Resources Theories

The JD-R model predicts organizational outcomes, through two main categories, namely, job demands and job resources (Demerouti et al., 2001). This model investigates job resources as personal, communal, and organizational resources, which can be exploited by employees to improve their respective physical and mental aspects (Demerouti and Bakker, 2011). Relatedly, job demands are defined as organizational requirements that require physical and psychological efforts for fulfillment. If individuals perceive job demands outweighing job resources, they will experience physical and cognitive costs in form of JS, exhaustion, and burnout (Schaufeli and Taris, 2014). SA as one of the emotional labor strategies to regulate felt emotions in accordance with demanded emotional display contributes to the increase in perceived job demands (Bakker and Heuven, 2006; Lee and Madera, 2019). SOC as a communal resource increases perceived job resources by individuals (Zou and Dahling, 2017).

The COR is a stress theory explaining individuals’ motivation to maintain and pursue tangible and intangible resources to attain or defend their goals (Halbesleben et al., 2014; Hobfoll et al., 2018). SA as a resource-depleting practice (Uy et al., 2017) activates the perception of resource loss among individuals, which consequently leads to JS and CW (Rafaeli et al., 2012; Zhang et al., 2016; Isoard-Gautheur et al., 2019). SOC is a resource addressing group-level spirituality at the workplace, which according to COR theory, can be exploited by employees to enhance their performance and wellbeing (Zou and Dahling, 2017). Thus, this study forms its theoretical foundation based on the aforementioned theories.

### Surface Acting and Job Stress

Empirical evidence suggests that SA brings stress to service workers (Grandey, 2003; Ogunsola et al., 2020). Many studies introduce SA as an unfitting emotional labor strategy leading to negative consequences such as JS and burnout (e.g., Li et al., 2017; Jeung et al., 2018). These findings are aligned with the fundamental theories of this study. According to the JD-R and COR theories, SA increases the perceived job demand and perceived loss/depletion of job resources and, consequently, will drive employees to higher levels of stress (Choi et al., 2019). Thus, we developed the following hypothesis:

*H1: There is a positive relationship between SA and JS.*

### Job Stress and Cognitive Weariness

Cognitive weariness is bound to chronic exposure to JS (Shirom and Melamed, 2006), and there are numerous studies suggesting a positive JS-CW linkage (e.g., Shirom, 2003; Rydmark et al., 2006; Bridger et al., 2011; Deligkaris et al., 2014; Isoard-Gautheur et al., 2019). This is also embedded within the aforementioned theoretical premises. Hence, the following hypothesis is established:

*H2: There is a positive impact of JS on CW.*

### Surface Acting and Cognitive Weariness

Surface acting involves inhibiting the expression of genuinely felt emotions, referred to as expressive suppression behavior (Yeung and Wong, 2020). Literature suggests emotion-regulation strategies involving expressive suppression behavior yield negative cognitive consequences (Richards and Gross, 2000). SA as a response-focused strategy consumes individuals’ cognitive resources, leading to cognitive exhaustion (Yagil, 2012). As a vital element, CW is regarded as the main dependent variable of this study. Therefore, the following hypothesis was developed:

*H3: There is a positive association between SA and CW.*

### Surface Acting, Job Stress, and Cognitive Weariness

Earlier literature stated SA, through increasing perceived job demands, contributes to JS. In addition, CW as a subdimension of burnout is generated by chronic stress. Thus, it is possible to infer that the effect of SA on CW is through JS. This inference is supported by literature suggesting the mediation effect of JS on the relationship between different dimensions of emotional labor and burnout (e.g., Choi et al., 2019; Theodosius et al., 2021). Hence, the following hypothesis is formulated:

*H4: The JS mediates the relationship between SA and CW.*

### Moderation Effect of Sense of Community

Many studies are investigating the moderation effect of SOC on employees’ wellbeing (e.g., Altaf and Awan, 2011; Saks, 2011; Kumar and Kumar, 2014; Zou and Dahling, 2017; Lata and Chaudhary, 2020). Chirico (2021) explained that during the COVID-19 pandemic, HCWs have experienced extremely high emotional loads because of witnessing the death of patients isolated from their loved ones. Chirico stated that lockdown situations and resulting social and economic issues along with fear of death and uncertainty about the future have complicated the emotional survival of healthcare professionals. The author concluded that spiritual resources are pivotal in order to face the current pandemic with less negative mental consequences. These findings are aligned with the foundational theories of this study considering SOC as a communal resource for fulfilling job demands regarding goals. Thus, the following hypotheses were shaped:

*H5: SOC moderates the relationship between SA and JS.*
*H6: SOC moderates the relationship between JS and CW.*

**FIGURE 1:**
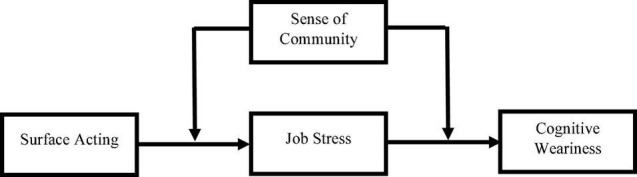
Research framework.

The hypothesized model of this study is illustrated in Figure 1.

## Methodology

### Data Collection and Sampling Design

The sample size was calculated using G*power software (Faul et al., 2007), and the resulting number is 126 (statistical power = 80%, effect size = 0.01, number of arrows pointing at CW = 6). Furthermore, the calculated sample size was compared with the recommendations of Hair et al. (2017) for the model (6 predictors, statistical power = 80%, Min *R*^2^ = 0.10, and α = 0.01), which is 179. Thus, any sample size above 179 should have adequate statistical power to draw valid conclusions. We used online questionnaires as the instrument for this quantitative and cross-sectional study. A pilot test was conducted with 35 nurses from a selected clinic to ensure the understandability and validity of items. The samples from the pilot test were not included in the data collection process. A total of 300 questionnaires were distributed among nurses working in different medical clinics and private hospitals in Tehran. With an 84.33% response rate, 253 responses were qualified for final analysis. Employing random sampling methods was not feasible for the authors at the time of conducting this study due to the COVID-19 pandemic. Therefore, we utilized a combination of non-probability methods of sampling to cover this limitation as much as possible. Accordingly, convenience and snowball sampling techniques were used to contact gatekeepers and Iranian nurses (Tongco, 2007). Furthermore, available nurses were filtered according to the inclusion criterion to select qualified respondents aligned with the context of this study (Etikan et al., 2016).

#### Inclusion Criterion

The criterion for inclusion was Iranian nurses, dealing with suspected or confirmed COVID-19.

### Respondents’ Profile

All 253 respondents were Muslim, with 87% women and 67% single (marital status). The average age among participants was 33.4 (*SD* = 5.9) years, and the average job tenure was 5.8 (*SD* = 6.1) years.

### Questionnaire Development

To minimize common method bias, all participants were assured confidentiality of responses (Podsakoff et al., 2003). Proximal separation based on Jordan and Troth’s (2020) instructions was practiced in the development of the questionnaire by asking respondents about their daily activities. In addition, focusing on SA and emitting deep acting will reduce the social desirability bias in nurses’ responses to this study survey (Larson, 2019; Taherdoost, 2019). Finally, a collinearity test was conducted and the variance inflation factor (VIF) scores did not exceed the cutoff value of 3.3 (Kock, 2015). Thus, there was no concern regarding the existence of common method bias in the collected data.

### Measures and Control Variables

All items measuring employed latent constructs are anchored on a seven-point Likert response scale. To measure SA, we adapted the seven-item scale originally developed by Grandey (2003). JS was measured using the short version of the Perceived Stress Scale with four items developed by Warttig et al. (2013). SOC was measured using Milliman et al.’s scale. In this scale, SOC is defined as a subdimension of workplace spirituality with seven items. CW was measured using five items of the Shirom-Melamed Burnout Measure (SMBM) developed by Melamed et al. (1999) as one of the subdimensions of burnout. The employed control variables were selected based on literature (potential demographic predictors of CW). Thus, marital status (Ortega et al., 2018), age, and job experience (Brewer and Shapard, 2004) were treated as exogenous variables regressed on CW (Becker et al., 2016).

### Analyses

Partial-least squares-structural equation modeling (PLS-SEM) was used to examine the hypothesized model. The justifications are (a) employment of latent variables in the model, (b) adequate statistical power with relatively small sample sizes, and (c) no concern regarding normality of distributions (Hair et al., 2017).

## Findings

### Assessing Measurement and Structural Models

The measurement model has been found acceptable since (1) outer loadings are above 0.708 (Hair et al., 2019); (2) values representing internal consistency including Rho A (Dijkstra and Henseler, 2015), Cronbach’s alpha (Diamantopoulos et al., 2012), and composite reliability (Jöreskog’s, 1971) are meeting satisfactory levels (0.7 < values < 0.9); (3) average variance extracted (AVE) values for constructs are above 0.5 representing the adequate convergent validity (Hair et al., 2017); and (4) values of the heterotrait-monotrait (HTMT) ratio as the most reliable measure of discriminant validity does not exceed the cutoff value of 0.85 (Henseler et al., 2015). Tables 1, 2 represent the assessment of the measurement model.

**TABLE 1:** Reliability and convergent validity of measures.

Constructs	Indicators	Outer loadings	Alpha	Rho A	CR	AVE
Surface acting	SA1	0.745	0.823	0.832	0.827	0.621
	SA2	0.899				
	SA3	0.915				
	SA4	0.781				
	SA5	0.757				
	SA6	0.913				
	SA7	0.841				
Job stress	JS1	0.814	0.884	0.892	0.885	0.749
	JS2	0.851				
	JS3	0.818				
	JS4	0.846				
Sense of community	SOC1	0.854	0.896	0.899	0.897	0.596
	SOC2	0.884				
	SOC3	0.894				
	SOC4	0.750				
	SOC5	0.902				
	SOC6	0.882				
	SOC7	0.819				
Cognitive weariness	CW1	0.899	0.832	0.844	0.835	0.703
	CW2	0.887				
	CW3	0.739				
	CW4	0.768				
	CW5	0.891				

**TABLE 2:** Heterotrait-monotrait ratio (HTMT).

	SA	JS	SOC
SA			
JS	0.624		
SOC	0.762	0.447	
CW	0.587	0.473	0.525

The structural model should meet the following requirements: (a) the normal fit index (NFI = 0.927) and the standardized root mean square residual (SRMR = 0.028) indicate satisfactory model fit (Henseler et al., 2014); (b) there was no concern with multicollinearity since values of inner VIF were below the 3 (Hair et al., 2019); and (c) values representing *R*-squared (in-sample predictive power) and *Q*-squared (predictive relevance) were calculated and meet satisfactory levels (Henseler et al., 2009). Table 3 depicts the structural model assessment.

**TABLE 3:** Structural model assessment and hypothesis testing.

Effects	Relations	β	*t*-statistics	ℱ^2^	Decision
**Direct**					
H1	SA → JS	0.324	5.203***	0.127	Supported
H2	JS → CW	0.424	6.841***	0.165	Supported
H3	SA → CW	0.218	2.971**	0.098	Supported
**Mediation**					
H4	SA → JS → CW	0.126	2.994**	0.034	Supported
**Interaction**					
H5	SA*SOC → JS	−0.142	2.247*	0.027	Supported
H6	JS*SOC → CW	−0.035	0.951	0.002	Not supported
**Control variables**					
	Marital Status → CW	0.152	2.471*		
	Age → CW	0.118	2.068*		
	Job tenure → CW	0.137	2.137*		
R^2^_JS_ = 0.38 / Q^2^_JS_ = 0.22					
R^2^_CW_ = 0.67 / Q^2^_CW_ = 0.43					
SRMR: 0.028; NFI: 0.927					

*0.05, **0.01, ***0.001.

### Hypotheses Testing

First, the findings of this study suggest a significant and positive relationship between SA and JS (β = 0.324, *t* = 5.203), supporting H1. Second, the results suggest a similar association between JS and CW (β = 0.424, *t* = 6.841), supporting H2. Third, similar results were found for SA and CW (β = 0.218, *t* = 2.971), supporting H3. Fourth, the findings propose a significant indirect effect of SA on CW through JS (β = 0.126, *t* = 2.994), thus, supporting H4. Fifth, the outcome of conducted analysis depicts that SOC significantly moderates the relationship between SA and JS through a buffering effect (β = − 0.142, *t* = 2.247). Therefore, the fifth hypothesis is supported. Finally, the results indicate that there is no significant moderation effect by SOC on JS-CW linkage (β = − 0.035, t = 0.951). Hence, H6 is not supported. The last finding corresponds with Crescenzo et al.’s (2021) results suggesting that there is no significant relationship between *transcendental orientation* (tendency to spirituality) and burnout dimensions. Table 3 provides the findings of hypotheses testing.

## Conclusion

### Findings and Theoretical Contribution

The results imply a significant effect posed on JS through SA. Embedded within the premise of JD-R and COR theories, this study provides a theoretical contribution in the context of the SA-JS relationship and, subsequently, JS-CW linkage. This not only supports the theoretical foundation of this study but also further provides a more thorough understanding of the underlying effects related to CW. In addition to what was noted, this study develops the theoretical understanding of mediation effects that are analyzed in the proposed model. Hence, this study contributes to both organizational psychology and healthcare management in terms of literature, and theoretical development. These findings extend the extant literature in a manner that opens a pathway for future researchers and agrees with prior studies (e.g., Ahmad and Ogunsola, 2011; Fontaine, 2018; Ogunsola, 2018; Ogunsola et al., 2020). Our findings suggest having employees who feel good within their workplace will have a collective effect exceeding one individual to others in the group and vice versa.

### Practical Implications

In the light of what was noted earlier, current findings provide practical implications that are derived from and are linked to its theoretical concepts. In this sense, a twofold implication for the managerial level and their strategies/actions regarding the retention of employees in the service sector is highlighted. First, emotional labor and its influence on wellbeing costs is emphasized and is to be taken into consideration by managers in the service sector, particularly those within the healthcare management industry (Hülsheger and Schewe, 2011; Zou and Dahling, 2017). Cognizance of managers is vital regarding the costs of emotion management and its requirements. This yields valuable, tangible, and positive outcomes in their respective firms (Grandey and Gabriel, 2015; Fontaine, 2018). Second, this study follows a string of research that emphasizes on positive outcomes of SOC within organizations. Although the workplace environment is commonly regarded as a secular space, spiritual concerns might be difficult for some managers to exhibit and reflect on (Chan-Serafin et al., 2013; Houghton et al., 2016). Consequently, this leads to an environment where individuals can sense spirituality and be encouraged to exhibit positive behavioral outcomes. As this study is written within the scope of Frontiers’ call for research “Spirituality in the workplace,” these findings contribute to the status quo of the extant literature. A manager can foster such an environment within the firm by establishing adequate and proper support for staff on an individual level. Our results are aligned with Karasek and Theorell’s (1990) expanded version of the job strain model, which includes social and communal support as a critical moderator of JS and psychological wellbeing relationship. The developers of this model note that job stressors can be reduced through workplace changes and the reorganization of production for less risk of stress is imperative (Crescenzo, 2016). There are a number of elements that can be used in this regard (see Rego and Pina e Cunha, 2008; Weinberg and Locander, 2014; Ogunsola et al., 2020).

Our results suggest organizations and subsequently their managers emphasize SOC. In this sense, initiatives and programs can be developed and designed to enhance SOC for employees and bridge the firm to its staff, customers, and society in a more profound manner. Such practices are essential for creating meaning at work through engaging with employees and developing their overall quality of life (e.g., Pratt and Ashforth, 2003; Saks, 2011; Ogunsola et al., 2020). Community-building initiatives provide a path for individuals within the firm to better establish interpersonal bonds and identify similarities (through cultural intelligence). These practices entail elements such as family-like dynamics at work, directed toward values. Notably, this direction exceeds merely profit concepts and enters the overall quality of life of employees (Ogunsola, 2018; Ogunsola et al., 2020).

### Limitations and Recommendations for Future Studies

Several constraints influenced the conduct of the current study while opening a pathway for future studies to include variables such as spiritual intelligence as a mediator for SA and DA negative linkage with OC. First, this study employed a self-report questionnaire for data collection. This poses a limit on capturing in-depth responses from respondents. Future studies can undertake qualitative methods to gain an in-depth understanding of the factors involved (i.e., interviews). Second, the data were collected in a cross-sectional manner, which limits the generalizability of findings. Future studies can obtain longitudinal data that can highlight changes and effects in a longer period. Third, interactions between job demand and resources can be better captured if reciprocal effects are assessed (see Schaufeli and Taris, 2014). However, this was not within the scope of the current study due to the cross-sectional nature of the data. Future research can avoid this issue by using longitudinal data for analyzing reciprocal effects among SA, SOC, JS, and CW.

Fourth, this study was limited due to restrictions caused by the COVID-19 pandemic, and, therefore, the probability sampling method could not be used. The validity of current findings can be tested through future studies using the probability sampling method. Fifth, nurses’ SA is considered through SOC as a context within the premises of JD-R and COR theories. As a consequence, personal resources can be included within the scope of these theories (e.g., self-efficacy, personality traits, and physical or cognitive conditions) (Xanthopoulou et al., 2007; Wang, 2020). Job demands, job resources, and personal resources can be incorporated in future studies to increase the generalizability of current results in the healthcare sector. Finally, SOC interventions can be experimented for enhancing JS and CW. In other words, SOC-building interventions within the workplace can be a variety of activities (e.g., workshops and different communication systems) to deliver the values of the firm to its employees thoroughly and ensure that individuals are aware of their value and importance and to achieve common goals for benefit of everyone involved. This can be tested in the context of the physical and psychological conditions of JS and CW.

## Data Availability Statement

The raw data supporting the conclusions of this article can be made available upon request by the authors.

## Ethics Statement

Ethical review and approval was not required for the study on human participants in accordance with the local legislation and institutional requirements. Written informed consent for participation was not required for this study in accordance with the national legislation and the institutional requirements.

## Author Contributions

PF: supervision. AS: analysis and writing. PZ: writing and editing. All authors contributed to the article and approved the submitted version.

## Conflict of Interest

The authors declare that the research was conducted in the absence of any commercial or financial relationships that could be construed as a potential conflict of interest.

## Publisher’s Note

All claims expressed in this article are solely those of the authors and do not necessarily represent those of their affiliated organizations, or those of the publisher, the editors and the reviewers. Any product that may be evaluated in this article, or claim that may be made by its manufacturer, is not guaranteed or endorsed by the publisher.
